# Annotations

**Published:** 1895-09-07

**Authors:** 


### ANNOTATIONS.

### London must give Degrees.

Should a university graduate love himself or his university best? The question is asked by reason of the attitude taken up by certain graduates of the University of London who have obtained medical degrees. There is a general consensus of opinion among able men that the prosperity of the London University would be increased, and that the status and culture of the medical profession would be improved, by a little relaxation of examinational stringency in the medical department at Burlington House. "No," say the present medical graduates; "for if that were done, the status of our degrees would be lowered, and that is what we are not going to allow." That is equivalent to saying that a university is made for particular individuals, and not for the maintenance and improvement of universal culture and progress. The point for the medical profession is this, that the M.D. degree has come to be considered the indispensable "hall mark" of a properly-educated doctor; and the doctor who does not possess that degree is looked askance at, as if his education and status were not of the best type to be found in the market. The argument is that the University of London should offer an average M.D. to the average student. At present it is contended that it offers only an honours M.D. to the honours student. As only a very small proportion of students take honours, by reason, not of their want of industry or capacity, but of the very great difficulty of taking such honours, it results that the ordinary student of the London schools must perforce go without his M.D. The grievance of the student is real; and the cause is foolish. No amount of examinational stringency can get more out of a man than Nature has put in; and no amount of obstinacy on the part of one British University can raise the M.D. degree above the general level conferred upon it by the British Universities as a whole. The effect, therefore, of the action of the London University is to deprive London students of a coveted distinction without increasing the value of that distinction. If London is to be as full of students as she ought to be, if she is to be a thriving mistress among medical schools instead of a mere "finishing academy," she must provide for the student of average industry and capacity an average M.D. degree.

### London Wants Students.

It is not the habit of Englishmen, more especially of English doctors, to "give themselves away." If it were, there are scores, perhaps hundreds, of clever men in London at the present time who would send the echoes of London's demand for students from one end of the metropolis to the other. The clever young doctors come to London—not all of them, of course, but a very large proportion of them—and they want work to do. The higher class of practitioners do not expect to get into much practice before the age of forty, though they have usually taken their degrees at the age of twenty-five, or even sooner. What are they to do between the ages of twenty-five and forty? To sit in a small room, reading, or writing an occasional newspaper article, and to wait for fifteen years for the patients, who seem as if they would never come, is about as melancholy a doom as any which even purgatory itself may have to offer. If all these clever young men could have two or three hours a day of teaching, instead of mere reading and writing, what an excellent thing it would be for their brains and spirits, and what indescribable advantages their students would reap from detailed tuition. That is one of the problems which London medicine has now to solve. Of teaching material there is a superabundance in all kinds. Of teachers, and competent teachers too, the name is legion. But many of them can find no teaching, even if they would do it for nothing. What is wanted is not merely more students, but improved systems and methods. In practically every subject in the medical curriculum, at least four times as many teachers could be profitably employed as are now at work. It is not mere lecturers of the old familiar type that are wanted, but tutors—tutors who will be content with half a dozen students each, and who will devote to that half-dozen all the energy and enthusiasm of eager and progressive minds. If a competent tutorial system could be established throughout all the medical schools of London, which would utilise all the teaching power that is available, but much of which is running to waste for want of use, the students at the London hospitals might be doubled in numbers within the next half-score years.

### The Doctor on Wheels.

So large a portion of a doctor's life is spent in going from place to place, that any promise of increased facilities of locomotion are to him a matter of prime importance. Few people have any idea how large a proportion of many a country doctor's gross income is eaten up in the stable, and anything which can lighten this grievous burden will undoubtedly be hailed by many a one with great joy. This possibility seems to be opening out in the direction of the petroleum-propelled tricycle. Sir David Salomons, in describing his investigations regarding "*voitures auto mobiles*" during a visit to Paris, says: "I saw a very pretty tricycle propelled by a tiny petroleum motor. Its weight is under 90 lbs., and the price is £52. The petroleum vapour is ignited by means of an electric spark. In order to set the machine in motion the rider mounts, turns a tap to admit petroleum, which at the same time turns on the electric current; he then propels the tricycle with his feet in the usual manner until he finds that the motor is working. Another handle is moved which releases the treadles, and the tricycle is then in full swing." This is just what the doctor wants. We do not believe that the ordinary familiar "doctor's trap" will be done away with, nor is it likely that medical men will cease to make the horse a companion and a friend in their daily journeyings. For the morning round, things will probably remain much as they are, but it is clear that a great deal of the expense of the doctor's stable depends on the irregularity of the work, and on the fact that, for weeks together, the horses, although unemployed, are costing none the less. On the introduction of the modern cycle, much was hoped from it, and it was thought that so handy a means of transport would be largely made use of for extra journeys. Medical men, however, work long hours, their responsibility is great, and, even as it is, they are weary before the day is done; and the added labour of a cycle is then more than they can bear. But a motor which will run itself, and will cost nothing while standing idle, is another thing altogether, and if the petroleum tricycle should take a practical form, and prove capable of mounting moderate hills, we see a prospect of much saving of labour and expense to many a hard-working country doctor.

